# Simple benchmarking method for determining the accuracy of depth cameras in body landmark location estimation: Static upright posture as a measurement example

**DOI:** 10.1371/journal.pone.0254814

**Published:** 2021-07-21

**Authors:** Pin-Ling Liu, Chien-Chi Chang, Jia-Hua Lin, Yoshiyuki Kobayashi

**Affiliations:** 1 Department of Industrial Engineering and Engineering Management, National Tsing Hua University, Hsinchu, Taiwan; 2 Washington State Department of Labor and Industries, Olympia, Washington, United States of America; 3 Human Augmentation Research Center, National Institute of Advanced Industrial Science and Technology, Tokyo, Japan; Tokai University, JAPAN

## Abstract

To evaluate the postures in ergonomics applications, studies have proposed the use of low-cost, marker-less, and portable depth camera-based motion tracking systems (DCMTSs) as a potential alternative to conventional marker-based motion tracking systems (MMTSs). However, a simple but systematic method for examining the estimation errors of various DCMTSs is lacking. This paper proposes a benchmarking method for assessing the estimation accuracy of depth cameras for full-body landmark location estimation. A novel alignment board was fabricated to align the coordinate systems of the DCMTSs and MMTSs. The data from an MMTS were used as a reference to quantify the error of using a DCMTS to identify target locations in a 3-D space. To demonstrate the proposed method, the full-body landmark location tracking errors were evaluated for a static upright posture using two different DCMTSs. For each landmark, we compared each DCMTS (Kinect system and RealSense system) with an MMTS by calculating the Euclidean distances between symmetrical landmarks. The evaluation trials were performed twice. The agreement between the tracking errors of the two evaluation trials was assessed using intraclass correlation coefficient (ICC). The results indicate that the proposed method can effectively assess the tracking performance of DCMTSs. The average errors (standard deviation) for the Kinect system and RealSense system were 2.80 (1.03) cm and 5.14 (1.49) cm, respectively. The highest average error values were observed in the depth orientation for both DCMTSs. The proposed method achieved high reliability with ICCs of 0.97 and 0.92 for the Kinect system and RealSense system, respectively.

## Introduction

Human body landmark location estimation has been introduced in many ergonomics applications for evaluating adopted working postures [1], performing gait assessment [2], clinical measurement [3], etc. Full-body landmark locations are important when estimating body segment lengths, joint angles, and joint displacements to calculate joint net moments using biomechanical models [4, 5] in ergonomics analyses. Performing these analyses often includes the implementation of anatomical segments defined by using a conventional optical marker-based motion tracking system (MMTS) to track the positions of skin-mounted markers. Unfortunately, MMTSs are often impractical for field applications because of their high-cost [6] and complex setup [7].

Significant developments have been made in optical approaches for image acquisition technologies, and many low-cost and portable depth cameras have been released in recent years. Depth cameras provide color channels (red, green, and blue (RGB)) and a depth channel (D). These sensors can capture and identify RGB images along with their per-pixel depth information in real-time [8]. Several techniques are used by depth cameras for object image acquisition and depth information identification. For example, time-of-flight (TOF) technology obtains depth information through the time delay between light emission and light detection (i.e., Kinect v2, Microsoft). Active stereoscopic technologies reconstruct a three-dimensional (3-D) shape based on triangulation and epipolar geometry theory from multiple cameras (i.e., RealSense D435i, Intel) [9]. Hence, depth cameras can be used in many applications for estimating distances between objects and acquiring 3-D data within a reasonable measurement range [10, 11].

With the advantages of being marker-less, depth cameras are also becoming increasingly popular for monitoring human movement and identifying 3-D joint positions in and outside laboratory environments [1, 5]. They may also provide an accessible alternative to MMTSs for ergonomics applications. Among them, many studies have been based on the Microsoft Kinect depth camera, and researchers have shown further interest in performing various evaluations to better understand its validity. For example, Xu and McGorry [5] indicated that a first- and second-generation Kinect camera yielded an average error range of 76 mm to 179 mm 84 mm to 161 mm, respectively, when identifying joint center locations of sixteen static postures during daily activities. Plantard et al. [12] reported that the average error value of computed joint angles based on available Kinect skeleton data was between 7.7° and 9.2° for performing ergonomic task assessments under work conditions. In addition, the frame error for gait analysis when using data from the Kinect device varied across gait parameters [13], while significant agreement and a high correlation were also found between the Kinect-based parameters and Vicon MMTS data for gait assessment [2, 14].

Most similar studies typically reported the validity of using the proprietary Kinect-specific skeletal model to obtain human motion data. However, for certain applications, the Kinect-specific skeletal model may not be adequate for some rigorous ergonomics assessments because it lacks clear anatomical definitions for some joints [5, 15] and has insufficient anatomical landmarks [12]. In addition, as this technology evolves quickly, analyses directly derived from the Kinect-specific skeletal model may eventually be superseded [6]. Moreover, other depth cameras are available on the market developed by various companies (Intel^®^, Asus, etc.). To ensure that the estimation of target locations provided by different depth cameras is trustworthy in intended applications, a systematic method for evaluating the validity of depth cameras that uses their raw depth and color (RGB) data output is needed.

Previous studies have developed and validated different methods for identifying 3-D poses based on raw depth camera data. For instance, Kobsar et al. [6] created a point cloud from a raw depth image obtained by the Kinect depth camera and then applied an iterative closest point algorithm to track the vertical displacement of the runner’s torso from the point cloud dataset. Abobakr et al. [1] trained a deep convolutional neural network to predict the human body joint angle and analyzed working postures in depth images captured by a depth camera. Although various validation results for advanced methods have been reported, for on-site ergonomics applications, an alternative benchmarking method that can be used simply by operators without computer expertise would be helpful.

Therefore, the goal of this study is to present a simple method for benchmarking the estimation accuracy of depth cameras in comparison with a reference MMTS using a novel alignment board. For demonstration, the proposed method is used to evaluate tracking errors in full-body landmark location measurements of two different depth cameras with different data acquisition techniques (time-of-flight and stereoscopic) for a static upright standing posture as an example. Direct comparisons between two types of depth camera systems and thirty-two landmarks are performed. In addition, the reliability of the proposed method is investigated in this study.

## Materials and methods

### Method for benchmarking the estimation accuracy of depth cameras

This method was developed for calculating tracking errors of depth cameras based on the coordinate data identified by the MMTS in a 3-D space.

#### Novel alignment tool between the coordinate system of depth cameras and the coordinate system of a marker-based motion tracking system

**Fig 1:**
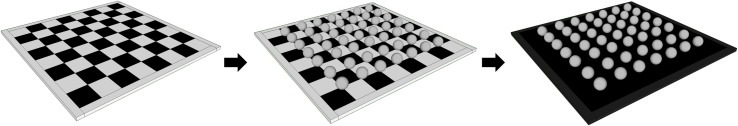
The idea of developing a novel alignment tool stemming from the concept of a checkered board. This study used the reflective markers which can be identified by MMTS in its coordinate system.

Alignment between different coordinate systems defined by different devices is an important step for comparing data from different camera systems. Generally, performing alignment lies in finding the correspondence between a sufficient number of known points in one coordinate system and its corresponding locations in another coordinate system. In the field of computer vision, researchers have proposed various methods to calibrate cameras. The method identified the known points based on two dimensional (2-D) objects, i.e., the intersection points of squares in a checkerboard, is one of the most popular techniques [16]. Therefore, this current study ideated the novel alignment tool based on the concept of it (Fig 1).

**Fig 2:**
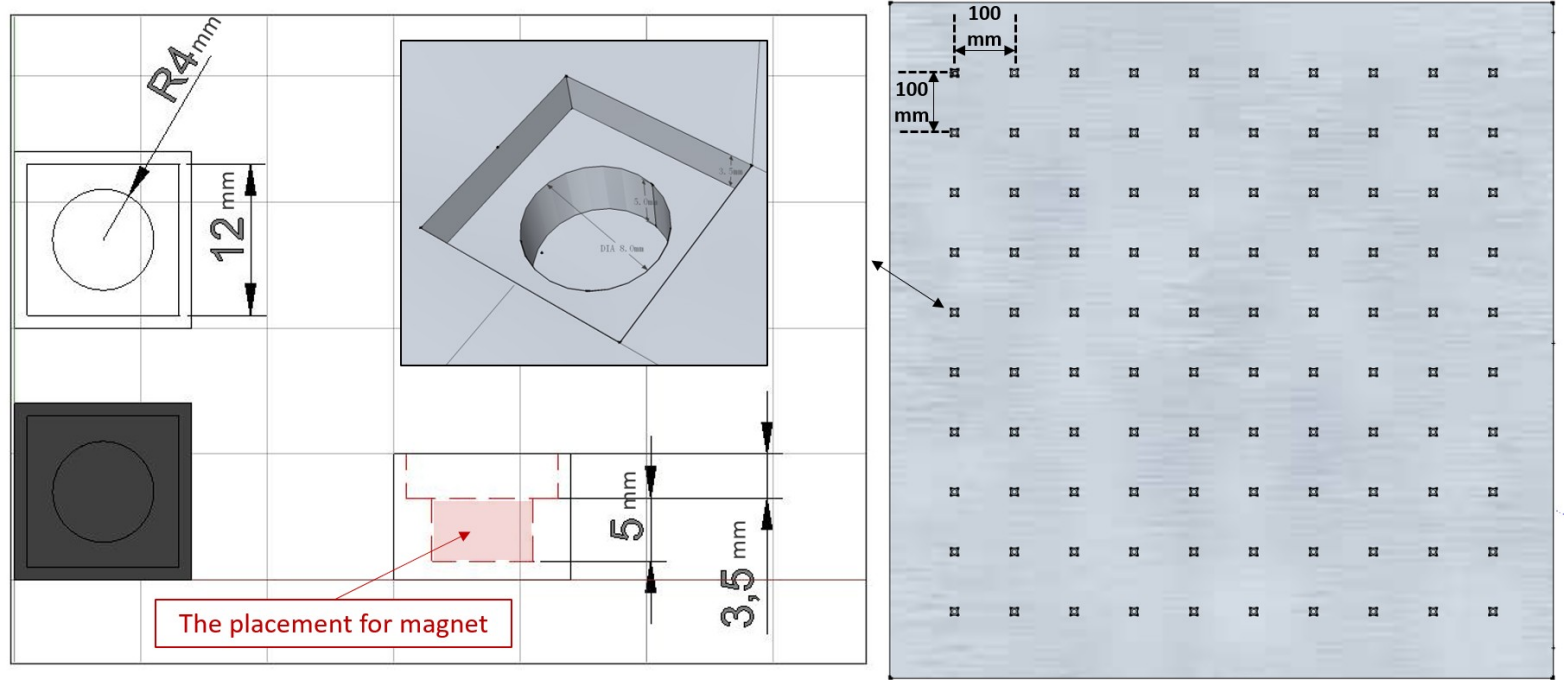
Sketch of the design for the alignment board. There are one hundred placements for the special reflective markers.

**Fig 3:**
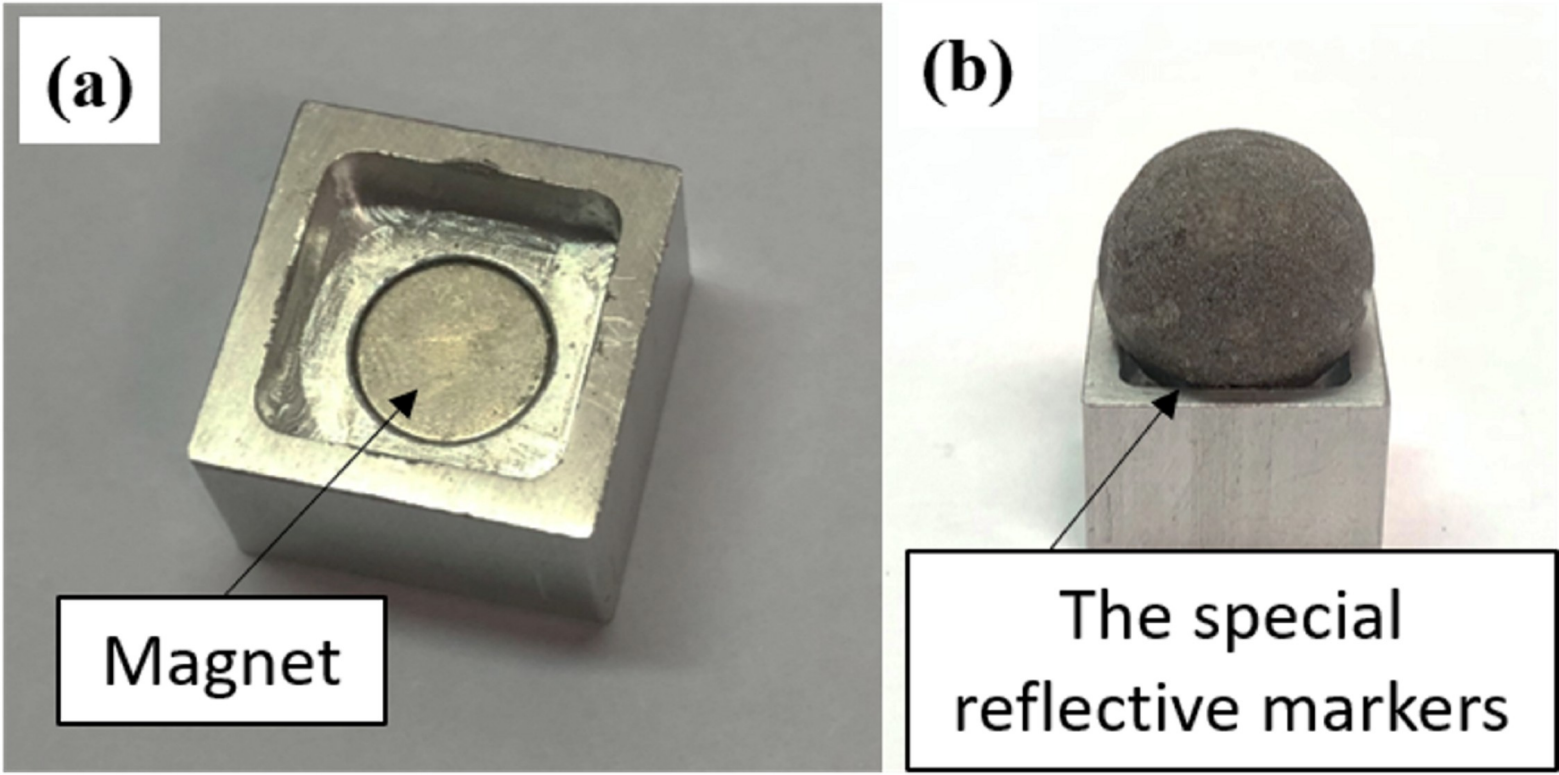
A sample of each of the holes that was used for special reflective marker placement. The special reflective markers (coated with reflective powder) are steel spheres, which can be fixed magnetically onto the alignment board.

A previous study indicated that the accuracy of a depth camera varies when the tracking target was placed at different locations and directions [17], therefore the proposed alignment board was designed to be of sufficient size, with a large number of marker placements. This board should allow a large tracking coverage of a depth camera, intended to reduce the error due to the potential measurement bias. A square aluminum alignment board (110 cm × 110 cm) was designed and fabricated (Fig 2). Previous studies utilized alignment objects ranged from an 11 × 8 checkerboard (a total of 88 quadrilaterals) [17] to a customized wooden wheel with a total of 16 sampling points [5]. Hence, we determined to choose the number of marker placements exceeding those used in the previous studies. On the plate, a 10×10-array of holes was drilled symmetrically for reflective markers placement. The distance between each marker placement was set at 10 cm, close to the length of smaller human body segments of interest, for example, the hand. The special reflective markers were steel spheres (diameter = 14.5 mm) coated with reflective powder, and they could be placed magnetically on the board (Fig 3).

**Fig 4:**
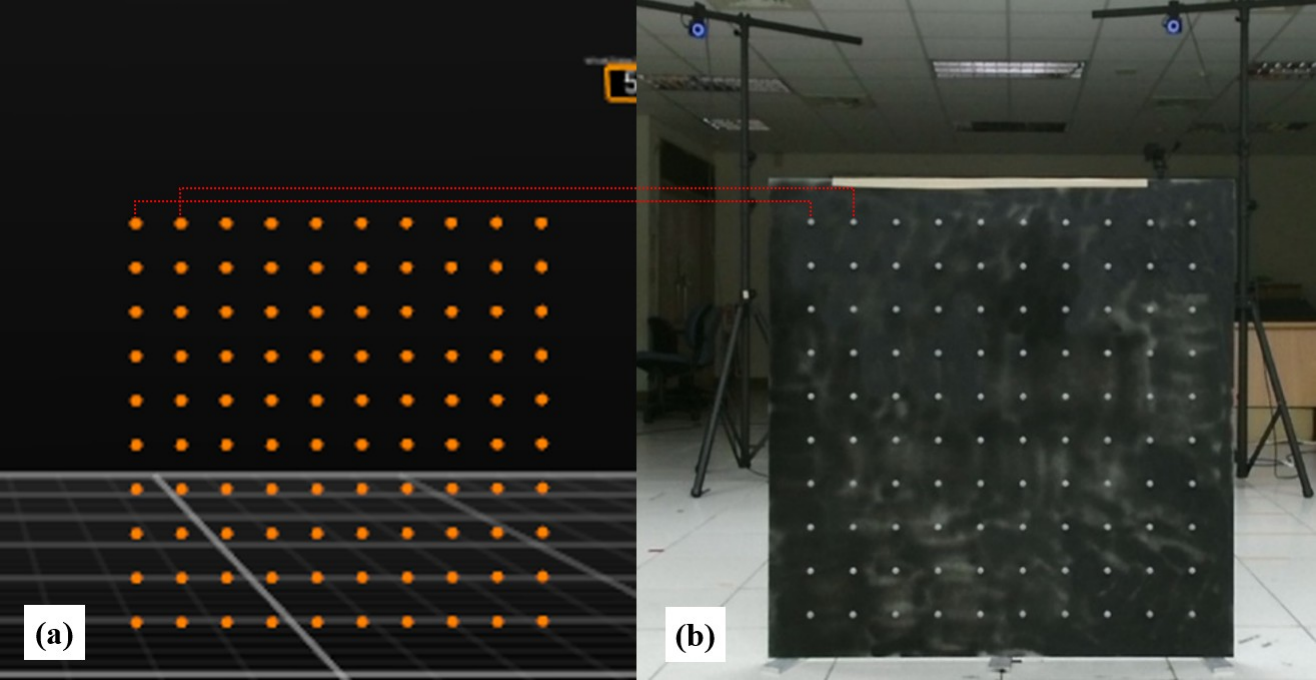
The special reflective markers can be tracked by the MMTS and DCMTS simultaneously. (a) MMTS view: the screenshot of the Motive software (OptiTrack Motion Capture System, NaturalPoint, Inc., USA) showed that one hundred markers were identified by the MMTS; (b) DCMTS view: the photo taken by a depth camera (the color space data (pixel, (x, y)) of each reflective marker on this picture was mapped onto camera space data (3-D space, (x, y, z))).

This design allowed the coordinate data of each special reflective marker on the alignment board to be recognized and captured by the MMTS (as reference) and depth cameras when there was no occurrence of occlusions. The transformation matrices for aligning the two coordinate systems could be generated based on the coordinate data of the markers on the alignment board as identified by each system (Fig 4).

#### Tracking error calculation

To illustrate the procedure of the proposed method, let symbols “M” and “D” represent coordinates x, y, and z in the coordinate systems (CSs) of the MMTS and depth camera, respectively. In addition, “t” and “b” are defined as the coordinate data of the measurement target and that of the special reflective markers on the alignment board, respectively.

To compare coordinate data of the depth camera and MMTS directly, a 3-by-3 rotation matrix (R_3,3_) and a 3-by-1 translation matrix (t_3,1_) that can be used to transform the coordinate data between the CSs are needed.

First, the coordinate data of two sets of corresponding reflective markers on the alignment board captured by the MMTS ${(x}_{b}^{M},y_{b}^{M},z_{b}^{M})$ and depth camera ${(x}_{b}^{D},y_{b}^{D},z_{b}^{D})$ were used to calculate the matrices R_3,3_ and t_3,1_. It follows that:

$${(x}_{b}^{M},y_{b}^{M},z_{b}^{M})={(x}_{b}^{D},y_{b}^{D},z_{b}^{D})\times R_{3,3}+t_{3,1}$$ (1)

Performing a coordinate transformation usually produces an estimated residual. The residual error (*Er_R_*) can be examined by calculating the average Euclidean distance between the original coordinate data determined by the MMTS $\left({x_{b}^{M}}_{i},{y_{b}^{M}}_{i},{z_{b}^{M}}_{i}\right)$ and the coordinate data from the depth camera after transformation ${(x_{b}^{D\to M}}_{i},{y_{b}^{D\to M}}_{i},{z_{b}^{D\to M}}_{i})$, where *i* is the *i*_th_ point on the alignment board and *n* is the number of reflective markers placed on the board:

$${(x_{b}^{D\to M}}_{i},{y_{b}^{D\to M}}_{i},{z_{b}^{D\to M}}_{i})=({x_{b}^{D}}_{i},{y_{b}^{D}}_{i},{z_{b}^{D}}_{i})\times R_{3,3}+t_{3,1}$$ (2)

$${Er}_{R}=\frac{1}{n}\left\{\sum_{i=1}^{n}{\left[{{(x_{b}^{M}}_{i}-{x_{b}^{D\to M}}_{i})}^{2}+{{(y_{b}^{M}}_{i}-{y_{b}^{D\to M}}_{i})}^{2}+{{(z_{b}^{M}}_{i}-{z_{b}^{D\to M}}_{i})}^{2}\right]}^{\frac{1}{2}}\right\}$$ (3)

After solving the rotation matrix and the translation matrix, they are used to convert the 3-D coordinate data of the measurement targets recognized by the depth camera ${(x}_{t}^{D},y_{t}^{D},z_{t}^{D})$ to the coordinate data based on the CS of the MMTS ${(x}_{t}^{D\to M},y_{t}^{D\to M},z_{t}^{D\to M})$:

$${(x}_{t}^{D\to M},y_{t}^{D\to M},z_{t}^{D\to M})={(x}_{t}^{D},y_{t}^{D},z_{t}^{D})\times R_{3,3}+t_{3,1}$$ (4)

Then, the error in the measurement of the coordinate data (Er) of each target location in 3-D space as measured with a depth camera can be calculated using the same concept of Euclidean distance via:

$$Er={\left[{\left(x_{t}^{M}-x_{t}^{D\to M}\right)}^{2}+{\left(y_{t}^{M}-y_{t}^{D\to M}\right)}^{2}+{\left(z_{t}^{M}-z_{t}^{D\to M}\right)}^{2}\right]}^{\frac{1}{2}}$$ (5)

### Demonstration

**Fig 5:**
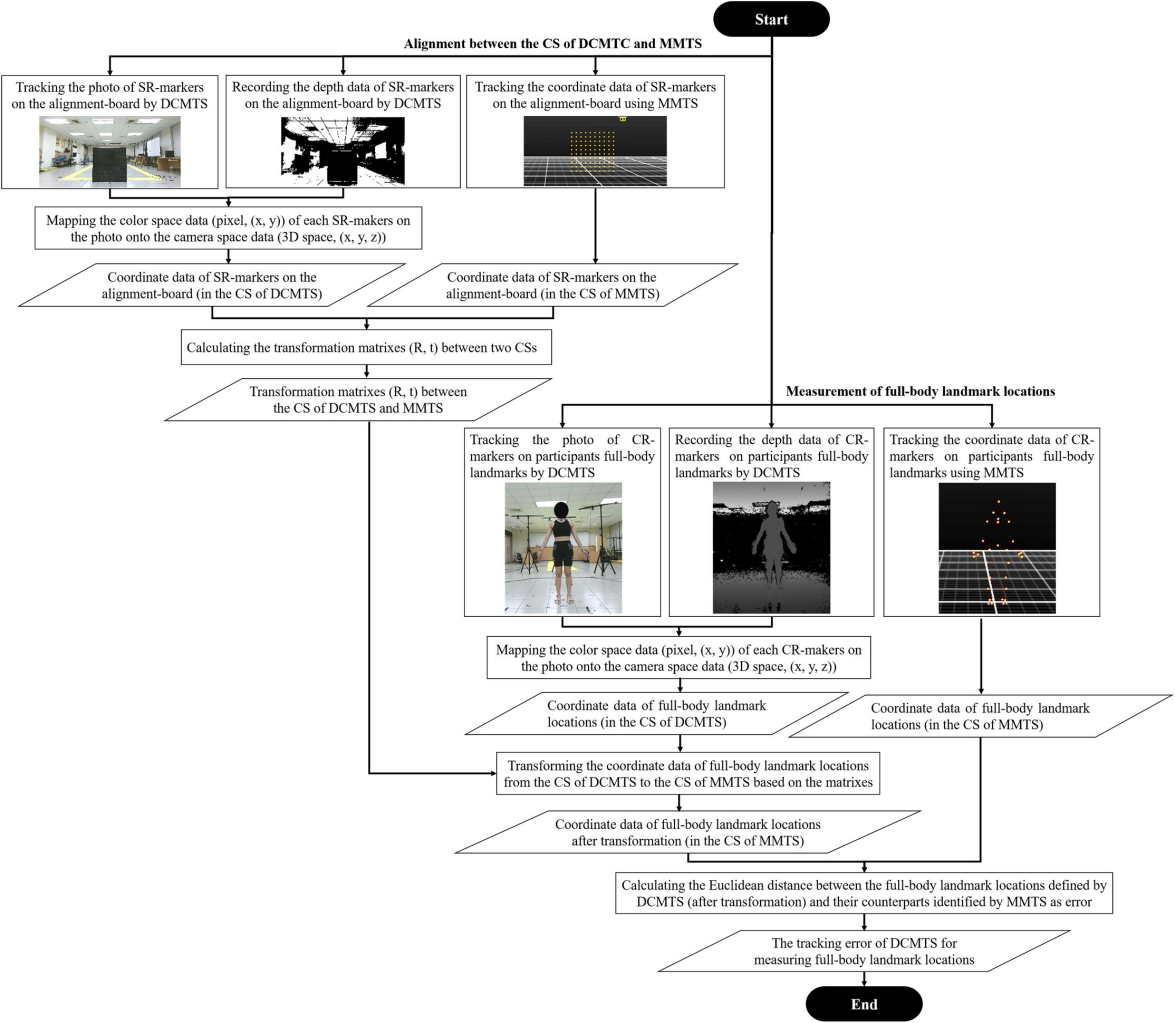
The flowchart for describing the use of the proposed benchmarking method. (MMTS: marker-based motion tracking system; DCMTS: depth camera-based motion tracking system; CS: coordinate system; SR-marker: special reflective marker; CR-marker: custom reflective marker).

As an example, this section describes the use of the method proposed in this study to assess the human full-body landmark location tracking error of two different depth camera-based motion tracking systems (DCMTSs), i.e., time-of-flight and stereoscopic. Here, body landmarks were defined as the measurement targets. Fig 5 shows the flowchart of this experiment.

#### Apparatus

An OptiTrack motion capture system (NaturalPoint, Inc., USA) sampling at 125 Hz was used as the gold standard. The second-generation Kinect (Microsoft, USA) with a frame rate of up to 30 fps was chosen to represent the time-of-flight technique. It consists of an RGB camera (resolution of 1920 × 1080 pixels) and a depth sensor (512 × 424 pixels). The second system chosen was RealSense D435i (Intel, USA) with a stereoscopic depth camera, a 1920 × 1080 pixel RGB sensor, and a depth sensor (1280 × 720 active stereo depth resolution, up to 90 fps). In this study, two different DCMTSs were defined (Kinect system and RealSense system). Two cameras of the same type placed at the front and back sides of the participants were combined to form a DCMTS. The distance between the two depth cameras was 5 m to track the target at the middle point, and the devices were placed at a height of 0.75 m. The full-body landmark coordinates were integrated based on the data output from one of the two depth cameras in a DCMTS.

The alignment board developed in this study was used as a tool for aligning the coordinate data between the two DCMTSs and their corresponding data defined by an MMTS.

#### Participants

The experimental protocol was approved by the local institutional review board of National Tsing Hua University in Taiwan. Three participants (age: 23.67 (2.08) years old, height: 1.68 (0.08) m, and weight: 63.67 (17.21) kg) provided written informed consent prior to participation in this study.

#### Experimental design and procedure

The same experimental protocols were used for the validation of the Kinect system and RealSense system.

First, the alignment board was placed in front of and facing each depth camera in the DCMTS and in the available field of view of the MMTS. In this experiment, 100 special reflective markers were placed on the alignment board. The locations of the markers on the board were recorded by the DCMTS and MMTS simultaneously.

**Fig 6:**
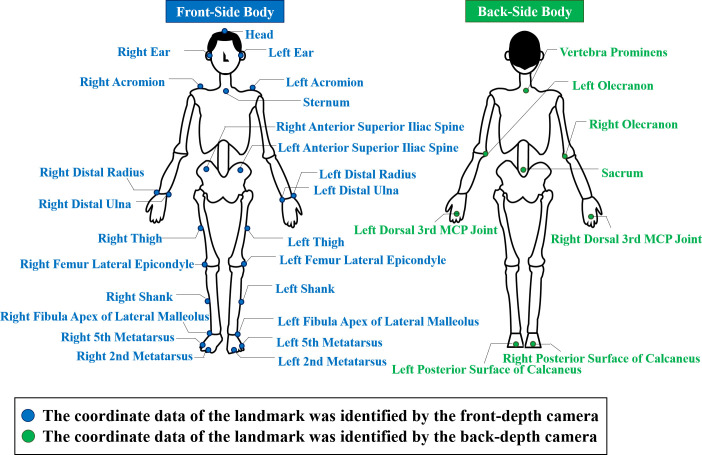
The target anatomical landmarks in this study.

Then, each participant was asked to stand in the middle of the DCMTS, face the front-camera, and hold a normal upright standing posture for approximately 2 s. Custom reflective markers that could be identified by the MMTS were attached to the thirty-two anatomical landmarks of the participants based on the tutorials of the professional biomechanics analysis software Visual3D (C-Motion Inc., USA). The target anatomical landmarks were chosen based on the “Rab Upper Extremity Model” [18] and the “Conventional Gait Model” [19–24], which can be implemented in Visual3D to compose a whole-body model, as shown in Fig 6.

#### Data collection

The coordinate data of the special reflective markers on the alignment board and the custom reflective markers attached to the thirty-two landmarks of the participants were tracked and identified by the MMTS using Motive (NaturalPoint, Inc., USA) software.

The color pictures (raw RGB data) with depth information (raw depth data) of the alignment board and the participants were taken by the DCMTS. For the Kinect system, Kinect software development kit (SDK) 2.0 was used to develop a customized application for recording and outputting coordinate data of the markers on the board and participants’ body landmarks. For the RealSense system, this study used Intel RealSense SDK 2.0 to write a program for obtaining the depth data of the reflective markers from the color picture.

The center points of the markers shown on the color pictures were manually selected, and their pixel-coordinate data were determined by an experimenter who performed several trials before formal data acquisition. Next, the color space data (pixel, (x, y)) were mapped onto the camera space (3-D space, (x, y, z)). This manual selection procedure was repeated twice by the experimenter based on the same dataset.

As shown in Fig 6, the landmark data on the front-side of the participants were taken by the front-depth camera (which the participants faced); the landmark data of the participants’ back-sides were obtained using the back-depth camera.

#### Data analysis

The x-axis represents the lateral orientation, the y-axis represents the vertical orientation, and the z-axis represents the depth direction of both depth cameras. The coordinate data of the special reflective markers on the alignment board tracked by the MMTS and each of the two DCMTSs were used to generate the transformation matrices and align the CS between them based on Eqs (1)–(3), respectively.

The coordinate data of thirty-two anatomical landmarks identified by two different DCMTSs were transformed into the CS of the MMTS using these matrices. The average Euclidean distance between the landmark locations defined by each DCMTS and their counterparts identified by the MMTS was used as an error. The difference between the coordinates based on the depth camera’s CS after transformation and the data from the MMTS of each axis (x, y, z) was also calculated using $\left|x_{l}^{M}-x_{l}^{D\to M}|,|y_{l}^{M}-y_{l}^{D\to M}\right|$ and $\left|z_{l}^{M}-z_{l}^{D\to M}\right|$, with *l* representing the meaning of each landmark. Based on the evaluation results of the proposed method (Eqs (4) and (5)), the estimation accuracy of each DCMTS could be benchmarked.

The average tracking error of two trials was used to compare the two different DCMTSs (Kinect system/RealSense system) when tracking full-body landmark locations.

In addition, the reliability of this method was assessed via intraclass correlation coefficient (ICC) based on two sets of tracking errors data of the thirty-two landmarks from two manual selection trials. The ICC value was evaluated using this order of agreement [25]: less than 0.5, poor; 0.5–0.75, moderate; 0.75–0.9, good and greater than 0.9, excellent. The standard error of the measurement (SEM) was also calculated. The SEM was defined as the standard deviation (SD) multiplied by the square root of the estimated reliability (Cronbach’s alpha in here) subtracted from 1.

## Results

For the alignments between the CSs of the DCMTSs and the corresponding CS of the MMTS, the average (standard deviation) residual errors (*Er_R_*) were 0.59 (0.04) cm and 3.77 (0.46) cm for the Kinect system and RealSense system, respectively, among the alignment steps.

**Fig 7:**
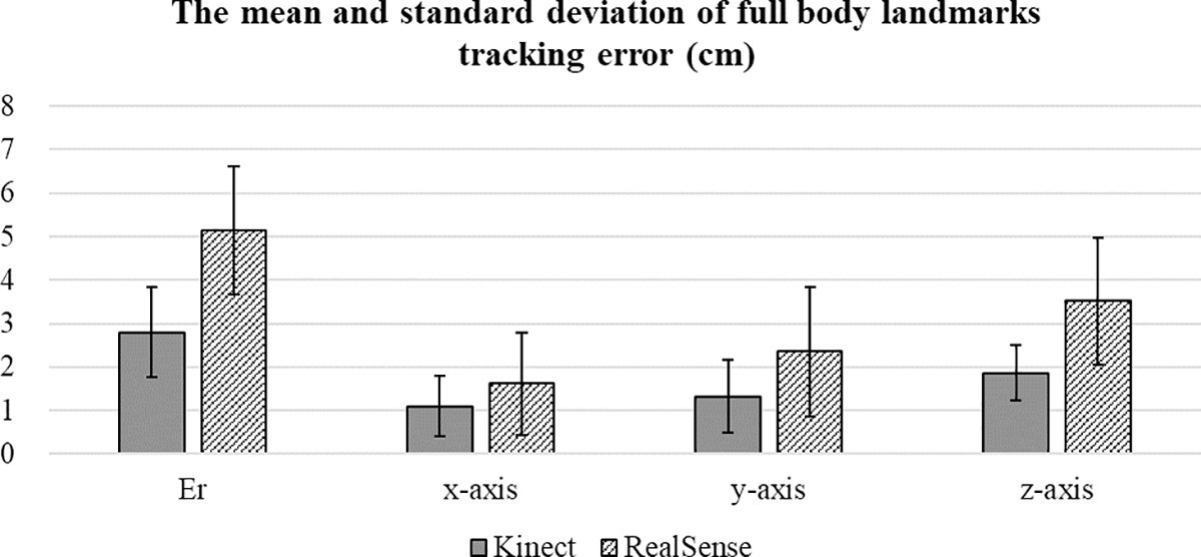
The average error value of the Kinect system and RealSense system in 3-D space.

**Fig 8:**
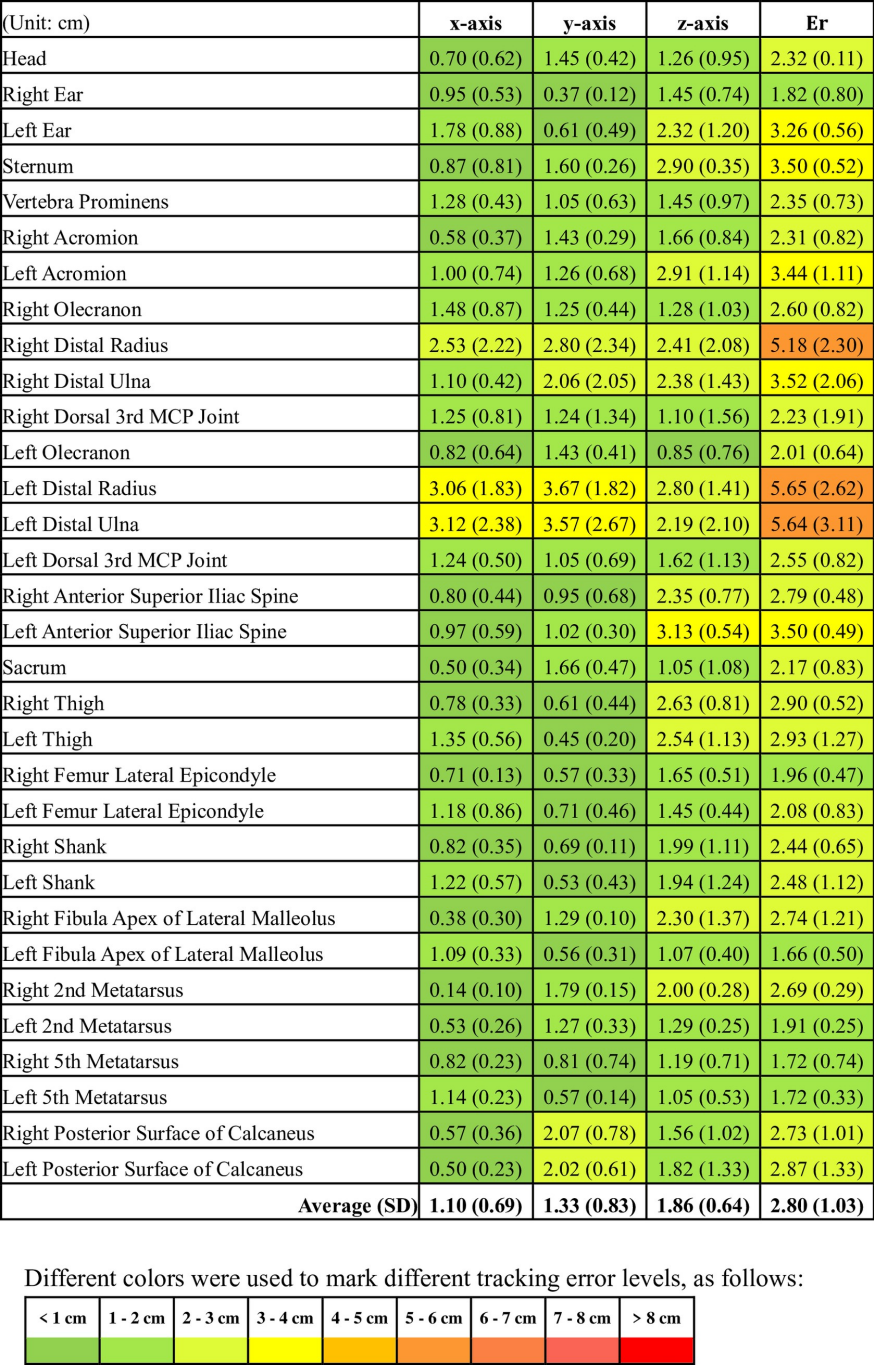
The mean (SD) of the Kinect system tracking error for full-body landmarks in 3-D space (Er) and each axis.

**Fig 9:**
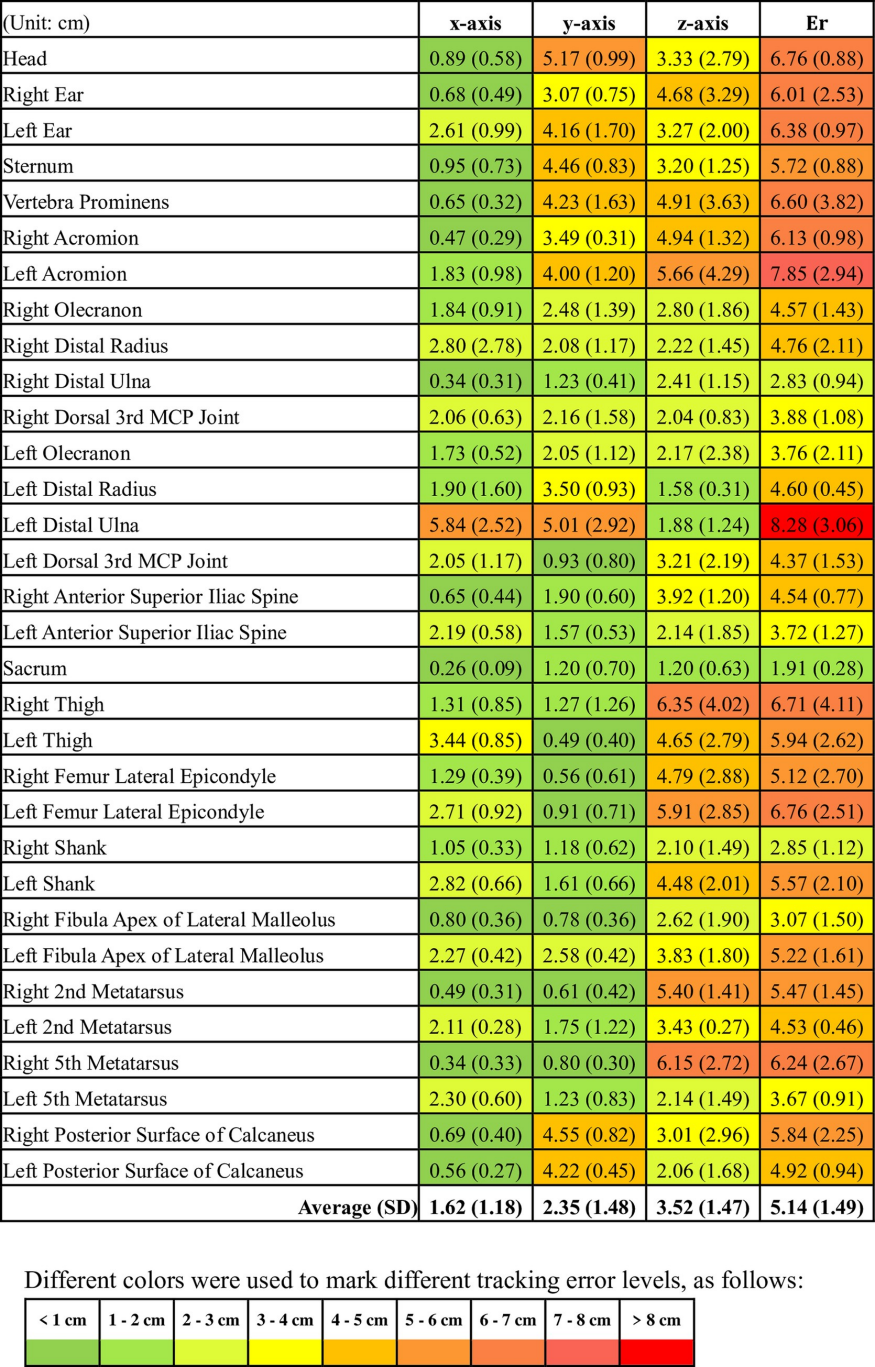
The mean (SD) of the RealSense system tracking error for full-body landmarks in 3-D space (Er) and each axis.

The average Euclidean distances of the three subjects and two trials for each target landmark of the Kinect system and RealSense system are presented in Figs 8 and 9, respectively. The range in the average error values (Er) was 1.66 to 5.65 cm and 1.91 to 8.28 cm, and the average error (standard deviation) of the full-body landmarks was 2.80 (1.03) cm and 5.14 (1.49) cm for the Kinect system and RealSense system, respectively, as shown in Fig 7.

For data tracking of three different axes using the Kinect system, the z-axis had a maximum average error value (standard deviation) of 1.86 (0.64) cm, and the x-axis had a minimum value of 1.10 (0.69) cm. Similar results were obtained for the data captured by the RealSense system, and a maximum average error value (standard deviation) of 3.52 (1.47) cm was observed for the z-axis. The x-axis had a minimum error of 1.62 (1.18) cm. Based on these results, overall, the Kinect system exhibited a better performance than the RealSense system in tracking full-body landmark locations for a static standing posture.

The method proposed in this study achieved high reliability between two measurement trials for benchmarking the tracking errors of thirty-two landmark locations using two different DCMTSs (Table 1). ICCs of 0.97 and 0.92 were found for the Kinect system and RealSense system, respectively, for evaluating the full-body landmark location tracking error. Reliability with an ICC higher than 0.9 was found in all error measurements of the x-, y-, and z-axes identified by the Kinect system (ICC: 0.95, 0.95, and 0.91, respectively). The landmark tracking errors defined by the RealSense system over two trials also showed ICC values higher than 0.9 in the x- and y-axes (ICC: 0.91 and 0.99, respectively), with the exception of the error measurement in the z-axis (ICC, 0.89).

**Table 1:** The reliability of the assessment of the estimation accuracy using the method proposed in this study for the Kinect system and RealSense system.

	Kinect system			RealSense system		
	ICC	95% CI	SEM	ICC	95% CI	SEM
Er	0.97	0.94–0.99	0.25	0.92	0.85–0.96	0.60
x-axis	0.95	0.90–0.97	0.23	0.91	0.82–0.95	0.52
y-axis	0.95	0.90–0.98	0.27	0.99	0.99–1.00	0.19
z-axis	0.91	0.82–0.95	0.29	0.89	0.79–0.95	0.71

ICC: intraclass correlation coefficient, CI: confidence interval, SEM: standard error of measurement

## Discussion

The present study developed an alignment board and a method for benchmarking estimation accuracy using depth cameras to capture full-body landmark locations in 3-D space. We propose the utility of raw depth/RGB data captured by depth cameras to measure the estimation accuracy of full-body landmark location identification. As previous analyses directly derived from the Kinect-specific skeletal model may eventually be superseded [6], we present a simple but systematic method that may be helpful for assessing the accuracy of alternative or upcoming depth cameras and is not limited to the two devices considered in this study.

According to the results (Figs 8 and 9), mapping 2-D pixels in a 3-D space using the Kinect system to obtain coordinate data of participant’s body landmarks exhibited an average error of 2.80 cm. In comparison, Xu and McGorry reported an average error value of 8.7 cm (the difference between the output data of a second-generation Kinect-specific skeletal model and an MMTS) over all major joints in a normal standing posture [5]. The current approach apparently can improve the accuracy acquired body landmark location data even when the same hardware device is used.

Additionally, based on the results observed in Fig 7, the Kinect system (with the time-of-flight technique) exhibited higher accuracy than the RealSense system (with stereoscopic technique) in identifying the thirty-two body landmark locations. This result indicates that for full-body landmark tracking, the Kinect system outperforms the RealSense system in the current setting. A similar result was found in the accuracy comparison between the Kinect and RealSense devices for measuring the fixed object dimensions [26]. The use of the time-of-flight technique seems to have better estimation accuracy than that of the stereoscopic technique. However, Chiu et al. [26] mentioned that the SDK of a RealSense device provides a wider range of options for altering the camera settings to adapt to different environments. The trade-off between accuracy and adaptability should be carefully considered to satisfy the user’s needs.

A new marker-less pose estimation library, OpenPose, was released for real-time multi-person 2-D pose estimation from an image or video [27]. However, the default 2-D skeleton output from OpenPose may not be sufficient for applications where body landmark locations in the 3-D space are required. Therefore, a previous study [28] developed a 3-D marker-less motion capture technique using OpenPose with multiple synchronized video cameras, and then evaluated its accuracy against the gold standard, an optical marker-based motion tracking system. Among all the error estimates based on the data in each of the axes (x, y, and z), only approximately 47% of errors were lower than 2 cm. The current study showed that there were 79% of the errors less than 2 cm for the Kinect system while a similar condition was observed from the RealSense system that only 47% of errors were less than 2 cm, compared with the gold standard system. Although the experimental conditions in this study were different from those in the previous study, it is still reasonable to believe that the utility of raw depth/RGB data captured by the Kinect system in this study can be an applicable alternative motion tracking tool.

The Table 1 results show that the proposed method for evaluating the full-body landmark estimation accuracy has excellent reliability for the Kinect system, with ICC values greater than 0.9 in all three axes. For the RealSense system, this method also exhibited excellent reliability for measuring the error in the x- and y-axes. For the z-axis, an ICC value of 0.89 was reached, which is slightly lower than 0.9 but remains within good agreement.

Other studies have used different parameters, such as joint angles [12, 29] and gait characteristics [30, 31], to test and determine the applicability of depth cameras in various fields. Regardless, our study used the distance directly calculated between the MMTS and DCMTS as the error because the coordinate data in 3-D space (x, y, z) are fundamental for estimating other spatiotemporal or kinematic parameters. Relying on advanced machine learning techniques, various methods for obtaining 3-D postural data without the help of a conventional marker-based motion tracking system have been developed and applied in improving motion assessment system [15], predicting joint load [32] and 3-D spinal postures [33]. However, the development and use of those methods usually require deep knowledge of those techniques and specific pose databases for certain required parameters. For example, using a deep neural network-based method to develop a 3-D lifting motion model would need a lifting dataset consisting of videos and corresponding 3-D joint information of various lifting tasks [34]. The validation results of those methods usually limited the applications to similar scenarios. In contrast, our proposed simple benchmarking method uses the depth camera’s raw depth and color data output, and directly calculates the distance between the MMTS and DCMTS for each fundamental body landmark location for universal usage.

The result shown in Fig 7 indicated that the error in the z-axis (depth orientation) was the main contributor when generating the tracking error of both DCMTSs in this study. A previous study [17] reported similar results: the first-generation Kinect had the highest error level of 1.1 cm in the depth direction across the entire tracking range. This finding should be considered in future experimental designs.

There were some limitations in the current study. First, the center location of each reflective marker in the color picture of the depth cameras was manually selected by only one experimenter. The interexperimenter variabilities associated with these determinations are unknown. The effects of variabilities among experimenters on the accuracy assessment should be further examined to understand if there is user-dependent error existed. Second, although different tracking distances and viewing angles may influence the accuracy of the depth cameras, this study only focused on developing a new method for assessing the accuracy and illustrating the method through demonstration experiments under a single condition based on the two selected depth cameras. In addition, because CSs differ among motion tracking devices, to directly compare their outputs, a step for aligning these CSs is important and necessary. The alignment board developed in this study, which has 100 special reflective markers, was used as a tool for aligning the CS of depth cameras with respect to the MMTS. It would be interesting to investigate whether the number and arrangement of the special reflective markers on the alignment board affects the result. Certainly, there is the time demand associated with the manual operations in this proposed method. This study intended to provide a systematics alternative benchmarking method for practitioners or operators to understand the accuracy of a depth camera for body landmark tracking before they conduct biomechanical or ergonomics analysis. The proposed benchmarking method is likely needed to be performed only once for an experiment to establish a depth camera system’s relative accuracy, and the time to carry this process out should be not significantly more than a typical calibration required for such motion tracking equipment. After this assessment, the user then can carry out the study. Therefore, this process is only a small portion of a whole study. Considering the value of understanding a system’s applicability before using it, we believe that it is a good trade-off. In future studies, with the emergence and advancements of computer vision techniques, using these approaches to automatically recognize special reflective markers on the alignment board may allow the aligning step to be performed efficiently. However, the idea of this proposed method should still be useful in assessing the estimation accuracy of various depth cameras, regardless of whether these assessments are performed manually by on-site operators or by specialists with advanced knowledge of computer vision-based algorithm development.

While the present study used postural data obtained at a single time point as a demonstration of the proposed benchmarking method, it did not limit the application of the proposed method to static poses. Given that a dynamic movement is composed of a series of individual static poses, that is, the video of dynamic movement recorded by a depth camera is the composition of serial images along the time. In principle, once the accuracy on each one of the images can be evaluated for a depth camera, the accuracy for dynamic movements can also be ascertained with further development. Similarly, the proposed benchmarking method should also be usable for studies that intend to use different postures as tracking targets to check the accuracy of the depth camera system. However, tracking different postures inevitably would encounter problems such as occlusions by the subject’s body segments or the surrounding objects when using camera-based systems. Further investigation on the effect of such challenges in the different complex levels of settings would be an important topic to expand the applicability of our proposed benchmarking method.

In addition, this study selected and evaluated two common depth cameras as representatives of the time-of-flight and stereoscopic techniques at the time of conducting this study. However, newer models of depth cameras will continue to be developed. The benchmarking results from this current study may not be able to represent the accuracy of all the new depth cameras. However, the proposed benchmarking method will still be applicable to other new depth cameras with similar principles in the future.

## Supporting information

S1 File Excel workspace including all needed raw data for the demonstration. (XLSX)
